# NET23/STING Promotes Chromatin Compaction from the Nuclear Envelope

**DOI:** 10.1371/journal.pone.0111851

**Published:** 2014-11-11

**Authors:** Poonam Malik, Nikolaj Zuleger, Jose I. de las Heras, Natalia Saiz-Ros, Alexandr A. Makarov, Vassiliki Lazou, Peter Meinke, Martin Waterfall, David A. Kelly, Eric C. Schirmer

**Affiliations:** 1 The Wellcome Trust Centre for Cell Biology and Institute of Cell Biology, University of Edinburgh, Edinburgh, United Kingdom; 2 Institute of Immunology and Infection Research, University of Edinburgh, Edinburgh, United Kingdom; University of Crete, Greece

## Abstract

Changes in the peripheral distribution and amount of condensed chromatin are observed in a number of diseases linked to mutations in the lamin A protein of the nuclear envelope. We postulated that lamin A interactions with nuclear envelope transmembrane proteins (NETs) that affect chromatin structure might be altered in these diseases and so screened thirty-one NETs for those that promote chromatin compaction as determined by an increase in the number of chromatin clusters of high pixel intensity. One of these, NET23 (also called STING, MITA, MPYS, ERIS, Tmem173), strongly promoted chromatin compaction. A correlation between chromatin compaction and endogenous levels of NET23/STING was observed for a number of human cell lines, suggesting that NET23/STING may contribute generally to chromatin condensation. NET23/STING has separately been found to be involved in innate immune response signaling. Upon infection cells make a choice to either apoptose or to alter chromatin architecture to support focused expression of interferon genes and other response factors. We postulate that the chromatin compaction induced by NET23/STING may contribute to this choice because the cells expressing NET23/STING eventually apoptose, but the chromatin compaction effect is separate from this as the condensation was still observed when cells were treated with Z-VAD to block apoptosis. NET23/STING-induced compacted chromatin revealed changes in epigenetic marks including changes in histone methylation and acetylation. This indicates a previously uncharacterized nuclear role for NET23/STING potentially in both innate immune signaling and general chromatin architecture.

## Introduction

The wide range of functions recently ascribed to the nuclear envelope (NE), the double membrane system surrounding the nucleus, indicates that it is a major signaling node for the cell [1], [2]. One of these functions appears to be the organization of chromatin. Indeed, gross structural rearrangement of chromatin is observed in a variety of diseases linked to the NE. In normal cells the majority of dense chromatin as inferred from electron microscopy (darker negative stained regions — the original definition of heterochromatin) occurs at the nuclear periphery. In cells isolated from patients with several NE-linked muscular dystrophies and cardiomyopathy this dense chromatin redistributes away from the NE [3]–[7] and similar chromatin distribution defects are observed in a mouse model for NE-linked cardiomyopathy [8]. Moreover, in cells from patients with NE-linked progeria, mandibuloacral dysplasia, and lipodystrophy the dense chromatin partly or completely dissipates [9]–[11]. In addition to these ultrastructural observations, changes in the distribution of epigenetic silencing marks were found in cells from patients with NE diseases and in tissue culture cells expressing disease mutations [12]–[14], leading to the idea that loss of this silencing function at the NE might alter gene expression to yield the disease pathologies. Indeed, changes in gene expression were found in patients with NE-linked muscular dystrophy and were recapitulated in a mouse model for this disease [15], [16].

The NE is thought to provide a principally silencing environment for several reasons. Early electron microscopy studies observed considerable dense chromatin at the nuclear periphery in resting lymphocytes that have little transcriptional activity while this dense chromatin largely dissipates in the activated state [17], [18]. Several individual genes have also been observed to move from the periphery to the nuclear interior as they become activated, including the *IgH* locus [19], the *Mash1* and *CFTR* genes [20], [21]. A more global profiling of genes and chromatin proteins in contact with NE proteins also supported the idea of the periphery as a generally silenced environment [22]–[25]. Thus disruption of this organization could have major and pleiotropic consequences for gene regulation.

The proteins mutated in diseases linked to the NE include both the nuclear lamins that form an intermediate filament meshwork underlying the inner nuclear membrane and several NE transmembrane proteins (NETs). Lamins themselves have been found to bind core histones [26]–[28], though no preference for modified histones was reported [29]. However, a more recent study found that an unprocessed form of lamin A could bind to heterochromatin protein 1 (HP1) alpha and that a farnesyl modification associated with a mutated form of lamin A in NE-linked progeria reduced this binding [13], though whether this binding also occurs with the processed lamins of other NE diseases has yet to be investigated. Some NETs, however, are known to associate quite specifically with silenced chromatin. For example, in yeast the nuclear membrane protein Esc1 interacts with Sir4 [30], while in mammals lamin B receptor (LBR) binds HP1 alpha and gamma [31] and preferentially binds to histone H3 carrying K9 tri-methylation that supports gene silencing [25], [32]. Additionally, the NET LAP2β can recruit HDAC3 to the periphery to deacetylate histones and thus maintain/increase silencing at the periphery [33]. Most of the NE-linked diseases with gross changes in heterochromatin have mutations in lamin A and the NETs LBR and LAP2β that affect heterochromatin have been reported to preferentially bind lamin B1; thus it is likely that other NETs exist that mediate the heterochromatin changes observed in most of these diseases.

One such NET that has been extensively investigated is emerin. Both mutations in lamin A and in emerin that cause Emery-Dreifuss muscular dystrophy result in the redistribution of electron dense chromatin away from the nuclear periphery [5]–[7]. Whereas LAP2β has been reported to bind lamin B1 [34], emerin has been shown to bind lamin A [35]. Although emerin has not been shown to bind specifically to chromatin with epigenetically silencing marks, it has been found to, like LAP2β, interact with HDAC3 [36]. Moreover, it has been found to bind to transcriptional repressors germ cell-less (gcl) and Btf [37], [38], which could either sequester the repressor away from targets in the nucleoplasm or potentially assist in recruiting their nuclear gene targets to the periphery. Indeed, it has been reported for both LAP2β and for emerin that their interactions with HDAC3 contribute to changes in spatial genome organization [39], [40].

To identify other NETs that might be involved in silencing/reorganizing chromatin either directly through compaction or through recruitment of silencing factors and thus possibly contribute to chromatin defects in NE-linked diseases, a panel of NETs that had been identified by proteomics [41] was screened for their ability to promote chromatin compaction when exogenously expressed based on a simple visual readout. This identified NET23 (gene name *TMEM173*), also subsequently called STING, MITA, MPYS, and ERIS [42]–[46] as a strong promoter of chromatin compaction. Several studies have now linked this protein to signaling events in innate immune responses upstream of the nucleus [42], [46], but have ignored its function in the NE. Nonetheless, chromatin remodeling, particularly through epigenetic modification, is associated with innate immunity [47], [48], and this is the first indication that NET23/STING might contribute to these signaling events in innate immune responses via chromatin remodeling. The compaction induced by NET23/STING was accompanied by changes in the epigenetic state of chromatin associated with silencing and the amount of compacted chromatin observed in a variety of untreated cells correlated with the endogenous levels of NET23/STING protein. Thus NET23/STING appears to add a chromatin remodeling function that contributes to establishing a particular chromatin organization in different cell types. This may indicate a novel nuclear function for NET23/STING in innate immune responses and may be involved in some of the chromatin changes that occur in wide-ranging NE diseases.

## Results

### A screen for NETs that promote chromatin compaction

**Figure 1:**
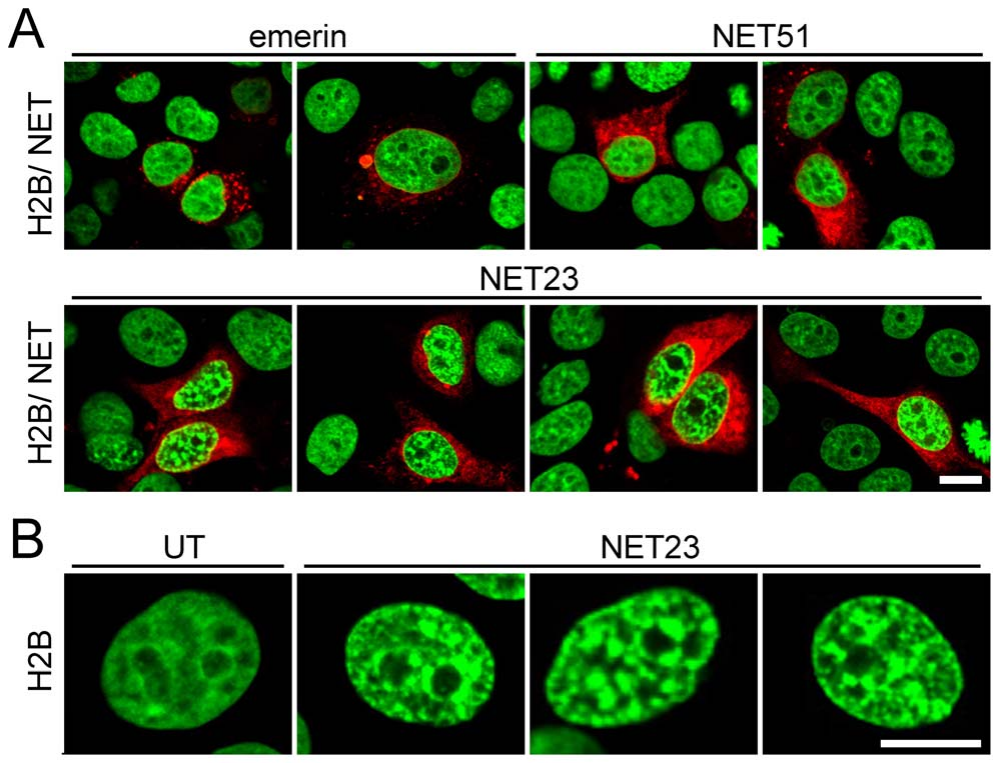
A screen for NETs that alter chromatin compaction. (**A**) 72 h post-transfection HeLa cells have no gross changes in distribution of H2B-GFP (green) when most NETs fused to mRFP (red) are exogenously expressed (e.g. emerin and NET51, upper panels). However, cells transfected with NET23/STING (lower panels) exhibit considerable chromatin compaction. (**B**) Zoomed images of chromatin in untransfected (left) and NET23 transfected cells. All images were taken using identical settings and all scale bars  = 10 µm.

To identify novel NE proteins that affect the compacted/condensed state of chromatin, 31 proteins that had been identified in a proteomic analysis of NEs were screened for their ability to promote chromatin compaction when exogenously expressed. These were cloned as mRFP and/or HA tag fusions [41], [44] and transfected into HeLa human cervical cancer epithelial cells stably expressing H2B-GFP [49]. Untransfected cells had little compacted chromatin based on the distribution of the marker, exhibiting an H2B-GFP pattern that was mostly diffuse with just a small increase in intensity around nucleoli (Figure 1B). Cells transfected with most NETs were still indistinguishable from the untransfected cells at 3 days post-transfection with no visible effects on the distribution of H2B-GFP labeled chromatin (Table 1 and Figure 1A, emerin and NET51, upper panels). NET30 and NET34 yielded a moderate increase in the amount of visually dense chromatin (Table 1), but expression of NET23 (gene name *TMEM173*) promoted a very strong increase in the amount of visually dense chromatin (Figure 1A, lower panels, and Figure 1B). As NET34 expressing cells looked generally unhealthy and smaller and NET30 expressing cells had a much weaker phenotype, subsequent studies were focused on NET23.

**Table 1:** List of proteins screened for effects on chromatin compaction.

NET	alt name	tag	fusion to	phenotype
4	Tmem53	HA	N-terminus	no effect
5*	Tmem201/Samp1	mRFP	C-terminus	no effect
8	LPGAT1	HA	N-terminus	no effect
13 (n)	SMPD4	mRFP	C-terminus	no effect on condensation, but many apoptotic cells
14 (n)	WDR33	HA	N-terminus	no effect
20	FAM105A	mRFP	C-terminus	no effect
23	Tmem173	mRFP	C-terminus	strong condensation
23	Tmem173	HA		strong condensation
24	ERIGIC1	mRFP	C-terminus	no effect
25	LEMD2	HA	N-terminus	no effect
26	Tmem14c	HA	N-terminus	no effect
29	Tmem120	mRFP	C-terminus	no effect
30	MOSPD3	mRFP	C-terminus	some condensation, but cells very sick
33	SCARA5	mRFP	C-terminus	no effect
34	SLC39A14	mRFP	C-terminus	some condensation and apoptosis
35 (n)	KIAA1967	HA	N-terminus	no effect
37	KIAA1161	mRFP	C-terminus	no effect
38	ALG2	mRFP	C-terminus	no effect
39	PPAPDC3	HA	N-terminus	no effect
43 (n)	NAT10	mRFP	C-terminus	no effect
45	DAK	mRFP	C-terminus	no effect
46	SLC22A24	mRFP	C-terminus	no effect
47	TM7SF2	mRFP	C-terminus	no effect
49 (n)	NOC4L	mRFP	C-terminus	no effect
50	DHRS7	mRFP	C-terminus	no effect
51	C14orf1	mRFP	C-terminus	no effect
52^t^		HA	N-terminus	no effect
54 (n)		mRFP	C-terminus	no effect
55	APH1B	mRFP	C-terminus	no effect
56	Dullard	HA	N-terminus	no effect
59	NCLN	mRFP	C-terminus	no effect
62	MCAT	HA	N-terminus	no effect

*shorter splice variant.

t truncated version, (n) indicates NETs that targeted poorly to the NE in these cells or appeared to be nucleoplasmic.

**Figure 2:**
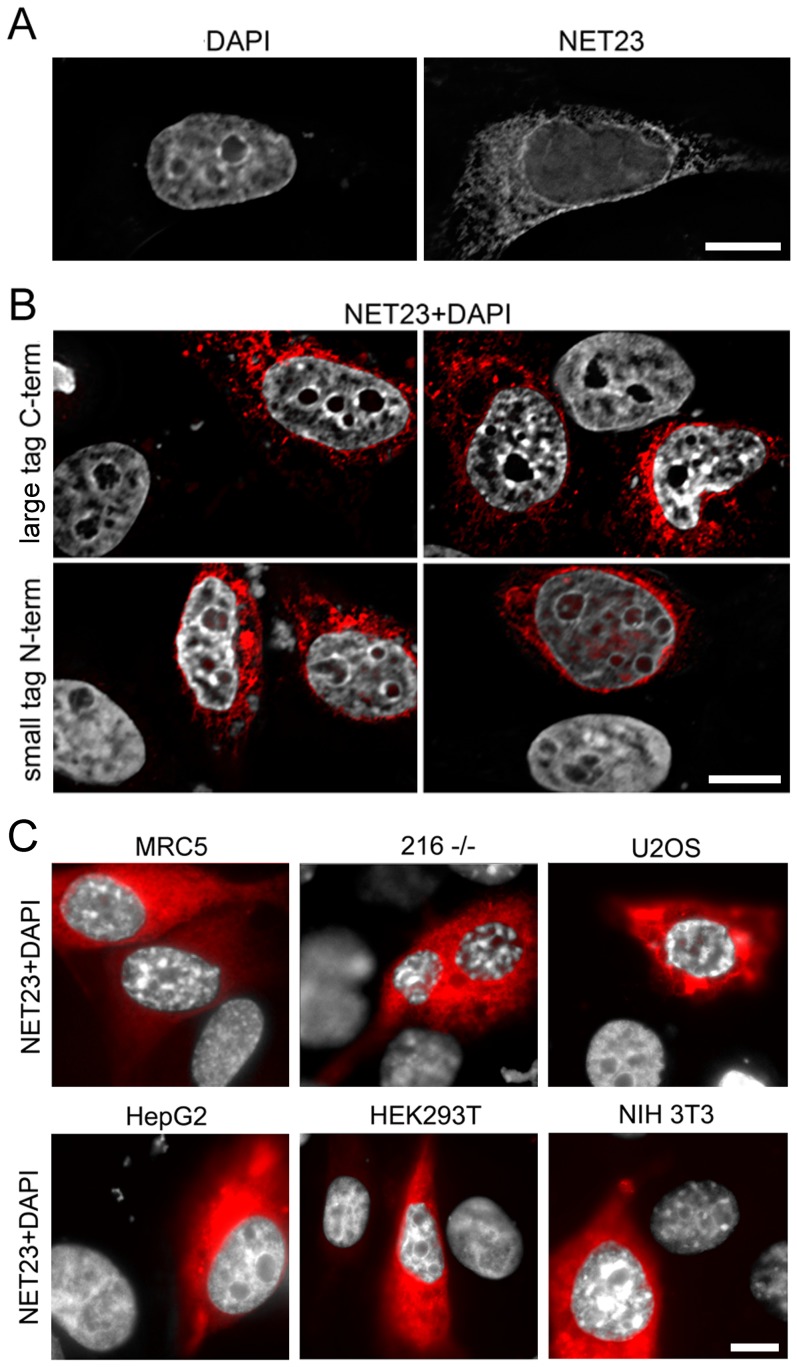
The NET23/STING chromatin compaction effect does not depend on H2B-GFP or the epitope tag used and occurs in a wide range of cell types. (**A**) Though at later times (72 h post-transfection) the compacted chromatin in the H2B-GFP HeLa cells was distributed throughout the nucleus (**Figure 1**), at 21 h post-transfection a large percentage of the compacted chromatin could be observed at the nuclear periphery. In this case, the compaction shown was visualized using DAPI to stain the DNA that yielded similar changes as observed for the H2B-GFP signal, indicating that outputs in subsequent experiments using other cell lines without the H2B-GFP could be compared. (**B**) The effect of NET23/STING is independent of the epitope tag used. NET23/STING with a large C-terminal mRFP tag (upper panels) or a small N-terminal HA tag (lower panels) both yielded the chromatin compaction phenotype in the H2B-GFP HeLa cells, again using DAPI staining to visualize the DNA. The NET is shown in red and the DAPI staining for DNA in grey. (**C**) The chromatin compaction phenotype of NET23/STING was not cell type dependent as the effect could be observed in MRC5 primary human lung fibroblasts, 216−/− lamin A knockout mouse embryonic fibroblasts, U2OS human osteocarcinoma cells, HepG2 human liver cancer cells, HEK/293T human embryonic kidney cells, and NIH3T3 mouse fibroblasts. Again, the NET23/STING is shown in red and the DAPI staining for DNA in grey. All scale bars  = 10 µm.

The compacted chromatin induced by NET23 expression was distributed throughout the nucleoplasm (Figure 1A and B). This might at first seem counter-intuitive because by definition a NET will be embedded in the membrane and NET23 has multiple predicted transmembrane spans so that it should in theory only be able to affect juxtaposed chromatin at the nuclear periphery. We postulated that NET23 might enzymatically act directly on chromatin or recruit factors to the NE that alter the chromatin or could additionally activate such factors at the periphery that could subsequently function throughout the nucleoplasm. However, it is reasonable that a function from the NE could be propagated throughout the nucleoplasm because studies using the Dam-ID method to determine globally the amount of chromatin at the nuclear periphery have indicated that a much higher percentage of the genome than can be physically present at the periphery at a given time interacts with the periphery, suggesting that many of the interactions are transient [50], [51]. Therefore the timeframe of 3 days post-transfection for fixation could have enabled a much larger percentage of the genome to interact at the nuclear periphery and so the H2B-GFP HeLa cells expressing NET23 were also viewed at 21 h post-transfection. Indeed, at this early timepoint after transfection the majority of compacted chromatin as determined both by the H2B-GFP signal (not shown) and by an increased density in the 4′,6-diamidino-2-phenylindole (DAPI)-stained DNA signal (shown) that mirrored the H2B-GFP signal was observed at the NE (Figure 2A).

NET23 has been independently reported on as both a mediator of innate immune signaling and apoptosis and separately named STING, MPYS, MITA and ERIS [42], [43], [45], [46]. As the STING name has been most widely used we will refer to the protein as NET23/STING henceforth. Although most reports on this protein have used fusions with a number of tags on both ends including HA, FLAG, GFP and RFP [43]–[46], one report stated that most tags interfered with its function [42]; so tags of different sizes at both ends of NET23/STING were tested. The compaction occurred independent of the tag placed on it or the position of the tag (Figure 2B). Nonetheless C-terminally tagged NET23/STING generally appeared to yield a stronger effect on chromatin compaction than N-terminally tagged protein, similarly to the report that N-terminal tags yielded less activity for experiments in innate immune signaling [42]. Furthermore, this is consistent with its topology that has previously been reported for a separate pool at the plasma membrane with the N-terminus in the cytoplasm [43], which would indicate for the nuclear pool the N-terminus being in the nucleoplasm. The nucleoplasmic region would need to interact with chromatin to modify it and so a tag on the nucleoplasmic N-terminus could in theory weaken this interaction.

The NET23/STING chromatin compaction effect was not due to a potential artificial interaction with the GFP labeled H2B molecules because it was also observed in multiple cell types not expressing the labeled [52] chromatin protein by using just DAPI staining for the DNA. Moreover, an increase in chromatin compaction as defined by the intensity of the DAPI signal was observed in all cell types even though they each had visually different basal levels and distribution of dense chromatin (Figure 2C).

### Chromatin compaction levels in different untreated cell lines roughly correlates with endogenous levels of NET23/STING

**Figure 3:**
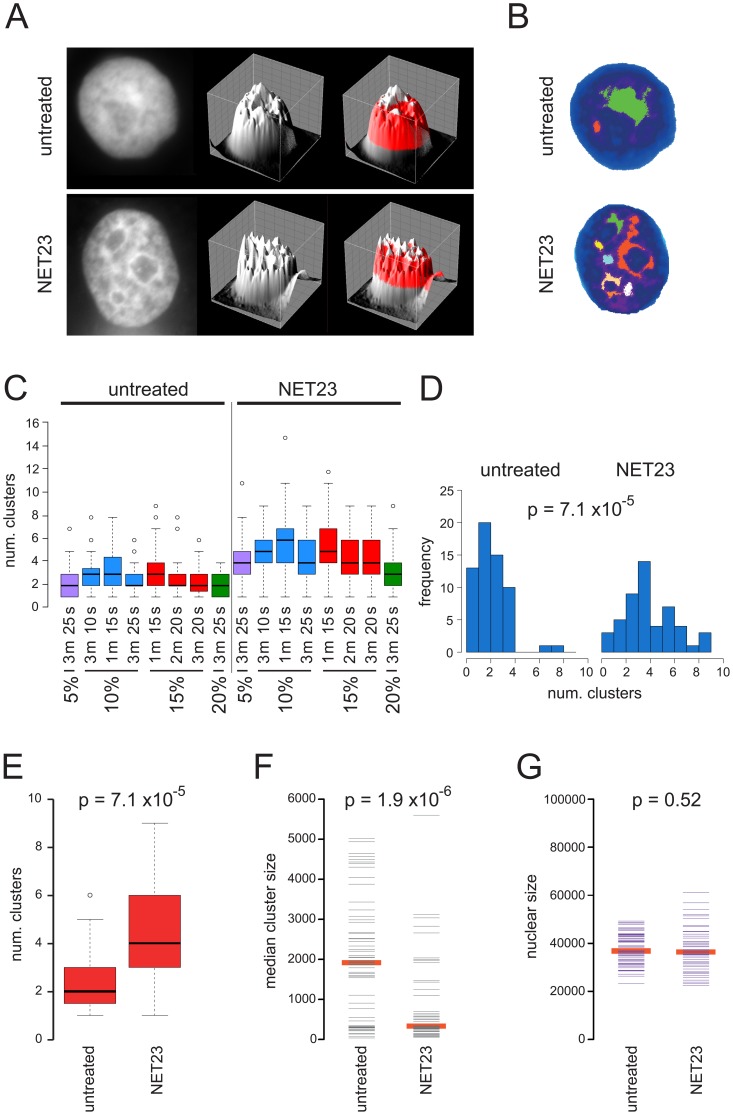
An algorithm for measuring chromatin compaction. (**A**) Pixel intensities from images obtained using identical microscope and camera settings were plotted topographically. A plane slicing through the topographic map at a particular percentage of the total intensity reveals only a small number of high intensity pixel clusters for untransfected cells while several high intensity pixel clusters can be observed for NET23/STING transfected cells. (**B**) Each high intensity pixel cluster for a particular plane in the cells shown in **A** is color-coded to visualize how accurately the algorithm distinguishes individual clusters. In setting the algorithm this step was used to optimize the parameters for numbers of pixels between clusters that would result in a merging of the clusters. (**C**) Several different parameterizations are able to distinguish between untransfected and NET23/STING transfected cells. A range of pixel intensity cutoffs for the plane are tested from 5–20% total pixel intensity (%). Also the number of pixels connecting clusters before merging them (m) and the minimum cluster size in pixels (s) were varied. This confirmed that the algorithm is robust and unbiased as statistically significant differences between the untransfected and NET23/STING transfected cells could be observed for nearly all parameters tested. (**D**) Histogram showing the shift in the distribution of the number of clusters between untransfected and NET23/STING transfected cells at the final parameters chosen: 15% signal intensities, 20 pixel minimum cluster size, and 3 connecting pixels required for merging. (**E**) Box and whiskers plot showing the distribution for the data in **D** and p-value calculated using a Kolmogorov-Smirnov (KS) test. (**F**) The same parameterization with plotting instead the cluster size medians. A similarly strong difference is observed with p-value calculated using a KS test. (**G**) Nuclear size was also measured for the cells analyzed and found to not change between the untransfected and NET23/STING transfected cell populations.

That the cell types tested had visually different endogenous levels of chromatin compaction raised the question of whether NET23/STING plays a role in this basal state. Indeed, NET23/STING was very highly expressed in lymphocytes and mouse cells that both visually tend to have high levels of chromatin compaction compared to most other cell types. To test this more directly, an approach was developed to objectively quantify the degree of chromatin compaction in the different cell lines so this could be compared to the endogenous levels of NET23/STING. In setting up the assay HT1080 cells derived from a human fibrosarcoma were used because they are known to have a lower basal level of epigenetic silencing marks and chromatin compaction and tend to maintain a reasonably stable genotype [53]. Nuclear DNA was stained with DAPI and imaged using identical microscope and camera settings (e.g. magnification, pixel size, exposure time, etc). High-density chromatin clusters were identified, and a number of metrics were calculated for them, such as number of clusters and size among others (Figure 3A). Nuclei with greater visual compaction based on the intensity of the DAPI signal after NET23/STING expression tended to have a larger number of smaller separate clusters of dense chromatin compared to untreated cells (Figure 3B). The method used three basic parameters. The main parameter is a signal threshold, to select pixels above a certain level. The two remaining parameters are minimum cluster size, to remove spurious isolated specks, and a merge parameter that controls how close two separate clusters can be before they are merged into one (see Materials and Methods for details). Various parameterizations were tested to confirm the method was able to distinguish between the two conditions (NET23/STING overexpression and untreated), including thresholds between top 5 to top 20 percentile of the DAPI signal, and a range of merge and minimum cluster size parameters (Figure 3C). For these control conditions across the entire range tested strong and clear differences could be observed with p-values using the KS test for all except the 20% signal intensity condition <1.94E-05. For further application the value of 15% signal intensities, 20 pixel minimum cluster size, and 3 connecting pixels required for merging was chosen with a p-value <1.1E-07. The differences in the distribution of numbers of clusters could be clearly observed using both histogram and box plots for this particular parameterization (Figure 3D,E). Other metrics were also checked such as area and size of clusters that also yielded significant p-values (Figure 3F). Since NET23-mediated nuclear compaction could be due to decreased nuclear area we have measured this parameter, but no difference in nuclear size was observed between the NET23-transfected and control cells (Figure 3G).

**Figure 4:**
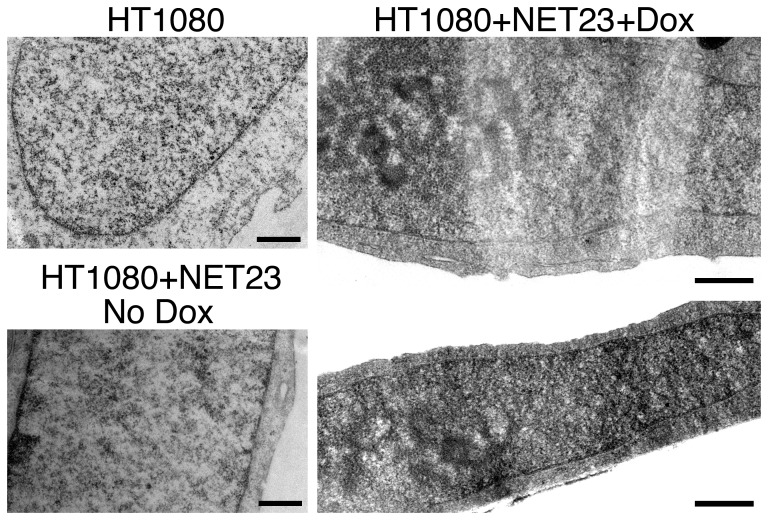
Chromatin compaction in NET23/STING overexpressing cells as visualized by electron microscopy. Panels on the right are for the HT1080 parent cell line and its uninduced progeny (HT1080+NET23 No Dox) carrying the integrated NET23/STING construct. Panels on the left are cells induced with doxycycline for NET23/STING expression for overnight prior to fixation for electron microscopy. Scale bars are 0.5 µm.

To determine the accuracy of the term compaction to describe the phenomenon being measured with the imaging algorithm, electron microscopy was performed on control HT1080 cells and HT1080 cells expressing NET23/STING. To ensure that all cells analyzed by electron microscopy expressed NET23/STING a stable-inducible cell line was generated. The degree of chromatin compaction as measured by denser stained areas of the nucleus by electron microscopy was similar between the parent HT1080 cells and uninduced HT1080 cells (No Dox) carrying the inducible NET23/STING construct (Figure 4, left panels). In contrast, induction of NET23/STING (+Dox) resulted in more electron dense clusters of chromatin both at the nuclear periphery and in the nuclear interior (Figure 4, right panels).

**Figure 5:**
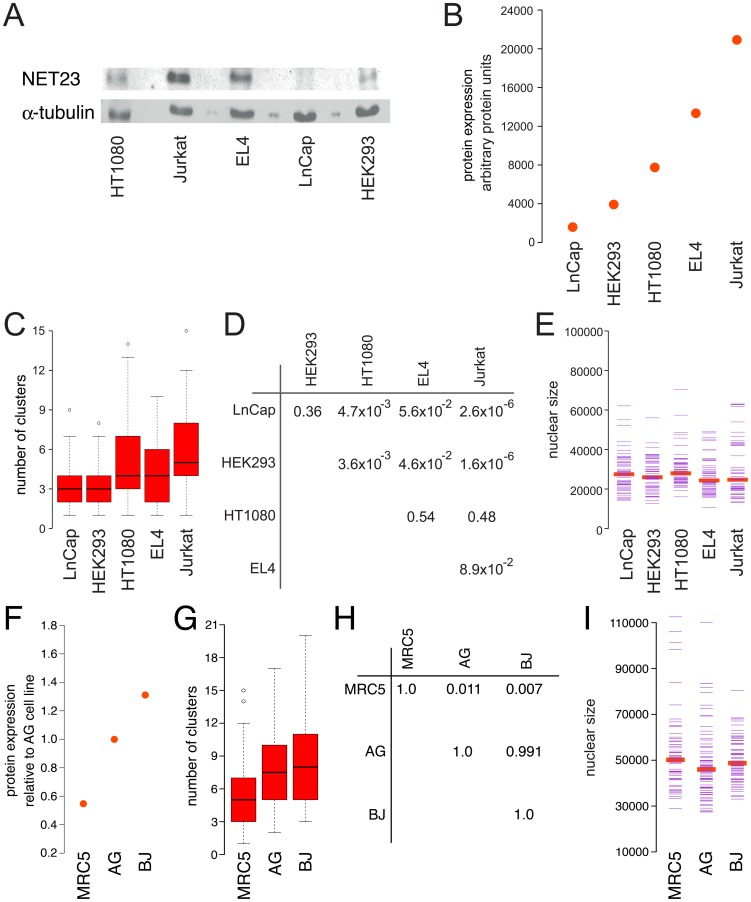
Endogenous NET23/STING expression correlates with levels of normal chromatin compaction observed in various cell types. (**A–E**) Transformed cell lines. (A) Western blot comparing levels of NET23/STING in different cell lines with α-tubulin used as a loading control. (**B**) Quantification of NET23/STING levels from three separate Western blots. The endogenous levels of NET23/STING have been corrected for α-tubulin levels and ordered from lowest to highest. (**C**) Using the algorithm described in **Figure 3** to determine endogenous levels of chromatin compaction in the same cell lines, similarly ordered, reveals a general trend that cells with higher endogenous levels of NET23/STING have higher levels of chromatin compaction. (**D**) Table showing p-values for C, comparing all possible combinations using KS tests. (**E**) Nuclear size was also tested for each cell line, finding no notable differences. All p-values for nuclear size using KS tests were>0.05 with the exception of comparing HT1080 and EL-4 cells (p = 0.039) and HT1080 and Jurkat cells (p = 0.003). (**F–I**) Primary cell lines. (**F**) Basal NET23/STING protein levels for three primary cell lines relative to the AG line. (**G**) Cluster algorithm to determine endogenous levels of chromatin compaction based on DAPI staining. (**H**) P values for comparing cluster number between the different cell lines using KS tests comparing each to the others. (**I**) Nuclear size measured for the three primary lines to ensure that all were similar so that this parameter could not influence cluster number results.

To test if NET23/STING could contribute to differences observed in the basal levels of chromatin compaction in different cell lines, NET23/STING levels were measured and chromatin compaction quantified based on the DAPI staining using the cluster algorithm in a variety of cell lines. As changes in nuclear size could in theory diffuse clusters, experiments investigating endogenous levels of NET23 utilized only cell types that had similar nuclear sizes (Figure 5). NET23/STING levels were measured by Western blot in five cell lines: HT1080, human fibrosarcoma cells; Jurkat, human immortalized T-lymphocytes; EL4, mouse lymphoma cells; LNCaP, human prostate adenocarcinoma cells; and HEK293, human embryonic kidney cells (Figure 5A). The amount of NET23/STING corrected against α-tubulin levels (Figure 5B) roughly correlated with the number of clusters measured in each cell type using the unbiased algorithm (Figure 5C). Significant p-values illustrate the general trend towards a higher level of chromatin condensation as the endogenous levels of NET23/STING increases (Figure 5D) and nuclear size was similar between all the cell types (Figure 5E) so that this trend could not be an artifact due to different nuclear sizes between the cell lines.

Several primary human cells were also examined as all of the above cell lines are immortalized cancer lines and so may have lost aspects of their parent chromatin organization. Only three of the primary lines tested had similar nuclear sizes and so could be considered: MRC5 lung fibroblasts, BJ foreskin fibroblasts, and AG dermal fibroblasts. Again the NET23/STING levels roughly correlated with the degree of compaction as measured using the cluster algorithm on DAPI-stained chromatin (Figure 5F–I). Thus NET23/STING may be involved in regulating endogenous levels of chromatin compaction.

### The timing of chromatin changes induced by NET23/STING

**Figure 6:**
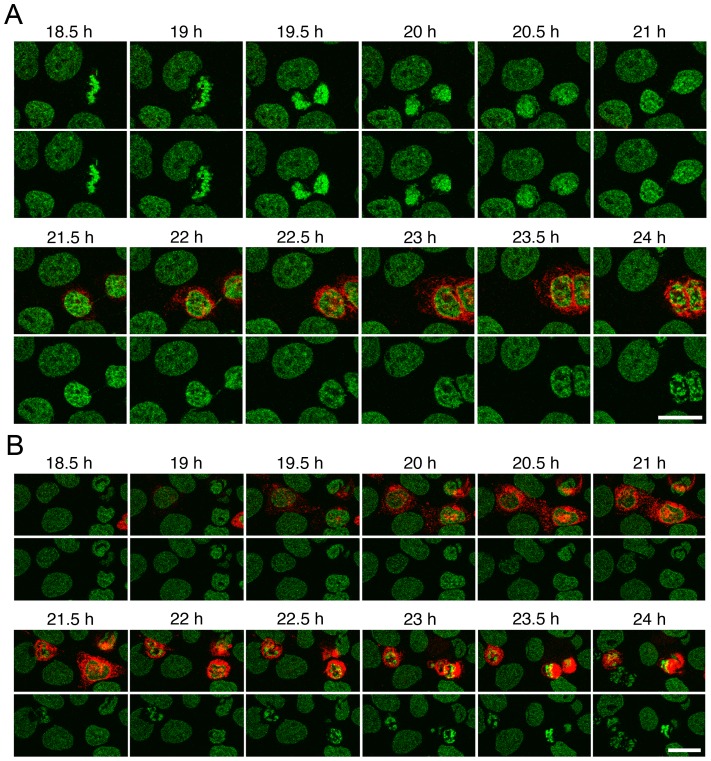
Live cell imaging of chromatin compaction reveals the process is fast and can lead to apoptosis. (**A**) Frames from movies of cells transfected with NET23/STING show the development of the chromatin compaction phenotype over time. The times shown are hours post transfection. Chromatin compaction begins at the nuclear periphery and then propagates throughout the nucleoplasm and considerable compaction is observed within 1 to 2 h from when the NET23/STING protein first appears. Note in the top movie that chromatin compaction looks distinct from that observed during apoptosis. (**B**) Many cells observed during live imaging yielded chromatin features and cell blebbing characteristic of apoptosis. From first appearance of NET23/STING to chromatin compaction and blebbing reminiscent of apoptosis typically took 2 to 3 h. All scale bars  = 10 µm.

Live cell microscopy with NET23/STING transfected cells revealed that the protein sometimes appeared shortly after telophase and sometimes much later (compare transfected cells in Figure 6A and 6B). An increase in chromatin compaction typically could be observed within 1 to 2 h following the appearance of the protein by fluorescence microscopy and the compaction was typically first observable at the nuclear periphery (Figure 6 and Figure 2A). Though many cells remained a long while with the chromatin in a condensed state visibly distinct from that of apoptosis (Figure 6A), a number of cells yielded chromatin compaction patterns characteristic of those associated with DNA fragmentation in apoptosis [52]; however, this occurred within 3 to 4 h following the appearance of NET23/STING (Figure 6B). This is very rapid for apoptosis because the DNA fragmentation/compaction stage typically is not observed even in the more rapid intrinsic apoptosis pathway until much later (e.g. 5–24 h for staurosporin-induced apoptosis, depending on cell type [54]). Though some cells previously expressing NET23/STING were observed to go through a successful mitosis in movies, many cells were observed to begin expressing the protein only after mitosis or in interphase where it was not possible to determine if the cells had previously expressed NET23/STING. As the cells were transfected with Fugene 6 and the liposomes maintained on the coverslips over the course of the experiment, it is possible that some cells fused with liposomes after dividing cells migrated closer to unfused liposomes on the coverslips. It is also possible that the changes to chromatin induced by NET23/STING make the cells more susceptible to laser damage in live cell microscopy. Therefore, it was important to quantify apoptosis separately.

### Exogenous NET23/STING expression promotes apoptosis

**Figure 7:**
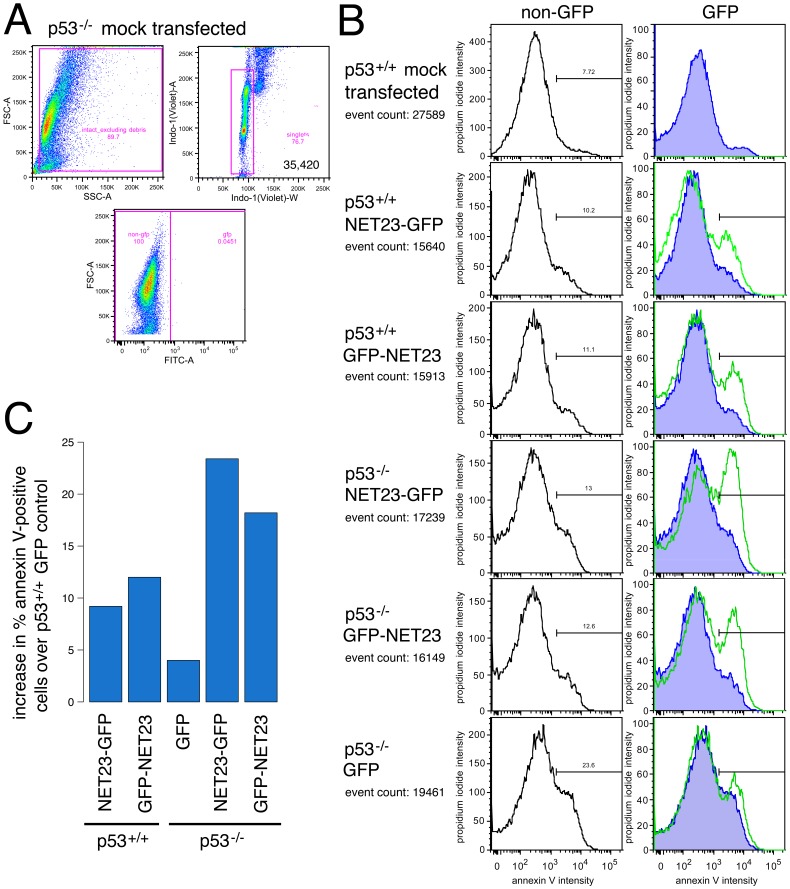
NET23/STING promotes apoptosis. (**A**) Gating strategy used for cells in B using forward and side scatter profiles to exclude debris followed by DNA content to determine intact singlet cells. The transfected population (expressing GFP) was identified by subsequently gating singlet cellular material on forward scatter versus GFP intensity. All cells in this experiment were analyzed at 44 h post-transfection. (**B**) The cells used to determine the gates were also stained for propidium iodide (PI; y-axis) and annexin V (x-axis). The traces in the left panels show the untransfected cells in the population and those in the right panels show the cells with GFP signal. The right-most green peak delineates cells with an annexin V signal of sufficient intensity to indicate cells undergoing apoptosis. As expected, for the mock-transfected culture essentially no GFP positive cells were identified and very few apoptosing cells could be observed. Expression of NET23/STING consistently increased the apoptosing population regardless of whether the tag was on the N-terminus (GFP-NET23) or the C-terminus (NET23-GFP) and the effect of NET23/STING did not require function of the master regulator p53 as apoptosis was induced in both wild-type (p53^+/+^) and p53 knockout (p53^−/−^) cells. Nonetheless, it is notable that the responses were very similar between the two NET23/STING constructs in the wild-type cells while the N-terminal tag showed a lagging apoptotic response in the p53 knockout cells. (**C**) The percentage of annexin V-positive cells is plotted after correction to subtract the number in the GFP control with the wild-type (p53^+/+^) cells. This is used as the correction for both cell lines to better indicate the effect of the p53 knockout itself on apoptosis induction.

To directly measure the percentage of apoptotic cells in the population without previous laser exposure stress, HCT116 human colon carcinoma cells transfected for NET23/STING with GFP fused at either the N- or C-terminus were fixed, stained with propidium iodide (PI) to measure all dying cells (PI only stains when the plasma membrane has been compromised) and annexin V for cells engaging early phases of defined apoptosis pathways, and analyzed by flow cytometry. Cells were gated on forward versus side scatter (FSC-A and SSC-A) to exclude debris before gating on DNA content to exclude aggregates to restrict analysis to intact singlet cells. Finally samples were gated on forward scatter versus GFP expression to distinguish transfected cells from non-transfected and very late stage apoptotic/necrotic transfectants (Figure 7A). This population is shown plotting the PI intensity against the annexin V intensity with non-transfected cells in each population in the left column and the transfected cells in the right column (Figure 7B). The right-most green peak indicates the cell population with a strong enough annexin V signal to indicate cells undergoing defined apoptosis. NET23/STING typically increased the percentage of cells in each of these three groupings by roughly 2–3 fold compared to the internal untransfected populations. The percentage of annexin V-defined cells undergoing apoptosis is plotted after correction for the GFP-transfected cells in the wild-type cell line (Figure 7C).

As the tumor suppressor protein p53 is commonly involved in inducing apoptosis in response to viral infections, we considered that NET23/STING might induce apoptosis via a p53-mediated pathway. Therefore the ability of NET23/STING to induce apoptosis was tested here in both wild-type HCT116 and HCT116 p53^−/−^ knockout cells. Apoptosis was induced in both cell backgrounds and to similar levels for the construct with GFP fused to the C-terminus of NET23/STING; however, intriguingly the construct with GFP fused at the N-terminus of NET23/STING exhibited a delayed response in the p53^−/−^ cells with more annexin V-positive and fewer PI-positive cells (Figure 7).

**Figure 8:**
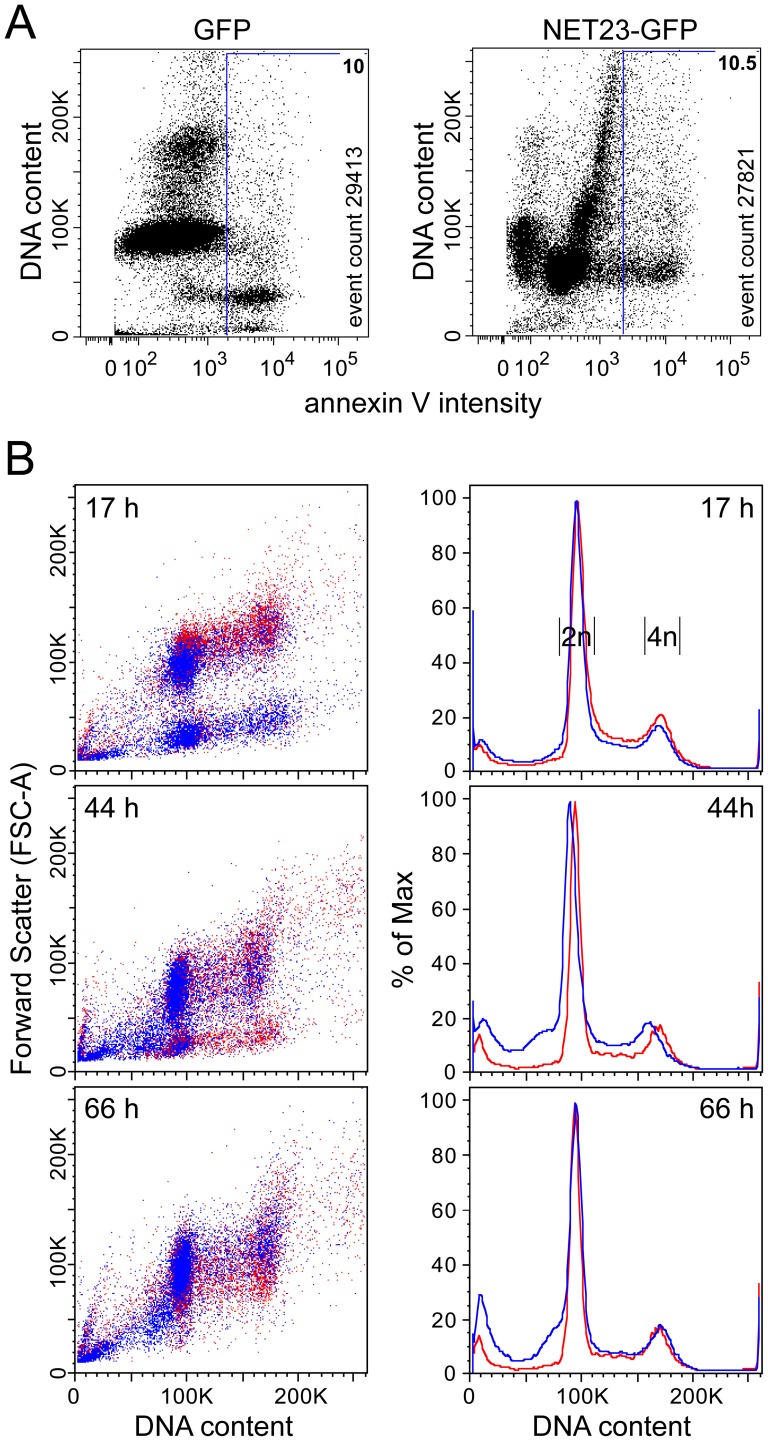
NET23/STING chromatin effects may set the stage for a transitional state between chromatin condensation and apoptosis. (**A**) Cells were taken at 23 h post-transfection and stained for DNA and the characteristic early apoptosis marker annexin V. GFP-transfected cells exhibit a normal distribution pattern with a large annexin V-negative G1 population (close to 100K) and smaller annexin V-negative G2/M population (close to 200K) and a small (∼10%) sub-G1 population that is mostly annexin V-positive. In contrast, at this early time post-transfection the NET23/STING-transfected population yields an aberrant distribution pattern with the main cell populations slightly lower than the normal G1 population, yet still slightly larger than the apoptosing sub-G1 population. This may reflect the process of chromatin condensation. (**B**) To investigate this population further, NET23/STING-transfected cells were analyzed over a timecourse from 17 to 66 h post-transfection. Over time the higher sub-G1 population can be observed to initially increase and then diminish as the smaller sub-G1 population increases. The density plots shown on the left plot DNA content against forward scatter to measure overall cell size/shape and thus likely give information about the shift in chromatin compaction, but these plots can be misleading about total numbers because of spots representing individual cells being printed on top of one another. In contrast, the cell cycle population plots on the right clearly show the total percentage of cells for the initial appearance of a higher sub-G1 population followed by its chasing into an apoptotic smaller/fragmented sub-G1 population.

When sampling NET23/STING transfected cells at earlier timepoints and instead plotting annexin V staining against DNA staining, an unusual distribution was observed (Figure 8A). At 23 h post-transfection, cells transfected with a plasmid expressing just GFP had a typical distribution pattern with most cells healthy in G1 and a smaller healthy population in G2/M. Only about 10% of transfected cells were sub-G1 with annexin V staining. By contrast cells transfected with NET23/STING fused to GFP at this early timepoint had similar levels of annexin V staining, but yielded a very unusual distribution pattern with most healthy cells running at a higher sub-G1 position based on DNA content. To investigate this further, NET23/STING-GFP transfected cells were followed at 17, 44, and 66 h post-transfection measuring both DNA content and cell size/shape by forward scatter (Figure 8B, left panels). Already at 17 h post-transfection the population of cells expressing NET23/STING included a group with a slightly reduced size, suggesting that this might reflect the chromatin compaction observed by microscopy. This population was however transient as it initially increased and then subsequently dissipated. The data were also plotted more quantitatively for DNA content (Figure 8B, right panels), indicating that while the larger sub-G1 population initially increased and then subsided the smaller sub-G1 population (close to the y-axis) indicative of dead/fragmented cells steadily increased. Furthermore, less than a quarter of the population of NET23/STING expressing cells in the intermediate compacted state stained with the Annexin V. Thus, we postulate that this higher sub-G1 state indicates an intermediate state where cells have compacted chromatin, but have not yet chosen to go down the apoptosis pathway. Nonetheless, that this state is transient while the smaller sub-G1 state steadily increases, suggests that most cells exogenously expressing NET23/STING eventually undergo apoptosis.

### Chromatin compaction occurs by a pathway independent of apoptosis

**Figure 9:**
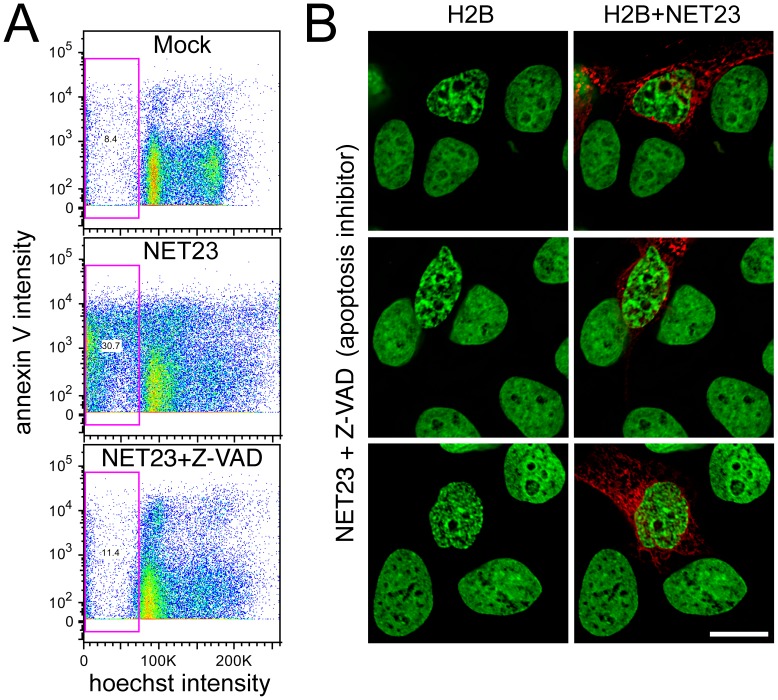
NET23/STING chromatin effects are independent of apoptosis and result in an increase in G2/M. (**A**) Untransfected cells (Mock), NET23-GFP transfected cells, and NET23-GFP transfected HT1080 cells treated with 20 µM of the pan-caspase inhibitor Z-VAD were stained for DNA content with the permeable dye Hoechst 33342 and the characteristic early apoptosis marker annexin V. The total sub-G1 population is gated (pink box) and anything above roughly 10^3^ should be positive for annexin V. Both the lower sub-G1 population and most of the annexin V staining of the NET23-GFP population are absent from the Z-VAD treated population. (**B**) HT1080 cells were similarly treated, fixed and stained for DNA for microscopy. Despite the blocking of apoptosis pathways with the pan-caspase inhibitor, the chromatin compaction still occurred in the NET23/STING transfected cells. Scale bars  = 10 µm.

Though the cells gave indications from the Annexin V staining that apoptosis was occurring in the NET23/STING transfected population, the rapid timecourse and initial appearance of the chromatin compaction were unusual for apoptosing cells. To test if the chromatin compaction phenotype was distinct from apoptosis pathways, populations of transfected cells were treated with the Z-VAD pan-caspase inhibitor or control buffer. The population was split and part was stained for DNA and immunofluorescence while the other part was stained with annexin V and analyzed by flow cytometry. The flow cytometry revealed that the Z-VAD significantly reduced apoptosis in the NET23/STING transfected population (Figure 9A). The microscopy revealed that the chromatin compaction phenotype was nonetheless still observed in the Z-VAD treated NET23/STING transfected population despite blocking apoptosis (Figure 9B).

### Chromatin compaction induced by NET23/STING is accompanied by epigenetic modifications

**Figure 10:**
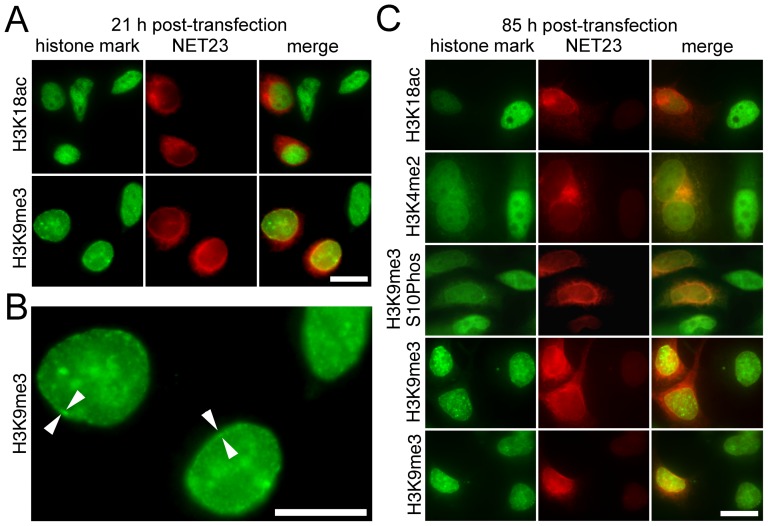
Epigenetic marks associated with heterochromatin coincide with the compacted chromatin at the nuclear periphery. Cells were fixed at various times post-transfection with NET23/STING and stained for various epigenetic marks, particularly the active marks histone H3 acetylation at lysine 18 (H3K18Ac) and di-methylation at lysine 4 (H3K4me2), the silent mark histone H3 trimethylation at lysine 9 (H3K9Me3) and the strongly repressed mark combined H3K9me3 and S10ph. (**A**) At 21 h post-transfection no change in the H3K18Ac was observed; however an increase in H3K9Me3 was already visually observable by immunofluorescence microscopy. (**B**) The chromatin compaction effects begin at the nuclear periphery. Higher magnification field from panel A. At 21 h post-transfection only a small amount of internal H3K9Me3 signal was observable while most was enriched at the NE (arrowheads). (**C**) At 85 h post-transfection both a loss of acetylation at K18 and methylation at K4 were observed indicating a general loss of active marks. At the same time a strong increase in methylation at K9 was visually observable by immunofluorescence microscopy, consistent with increased silencing. The H3K9me3 was seen throughout the whole nucleoplasm though some concentration at the NE could often be observed. However, the stronger repression mark H3K9me3 combined with S10ph was actually reduced in the NET23/STING transfected cells. All scale bars  = 10 µm.

As the chromatin compaction phenotype was independent of apoptosis and innate immune responses are often accompanied by epigenetic modification of chromatin [47], the observed chromatin compaction induced by NET23/STING might be mediated by epigenetic silencing mechanisms. To test for this cells were stained with antibodies to epigenetic marks at 21 and 85 h post-transfection. Antibodies specifically recognizing acetylation on histone 3 lysine 18 (H3K18ac — a standard mark for active or poised genes [55]) or tri-methylation on histone 3 lysine 9 (H3K9me3 — a standard mark for silenced genes [55]) were used. At 21 h post-transfection (Figure 10A) a small increase in H3K9me3 was observed compared to the adjacent untransfected cells, mostly concentrated at the nuclear periphery (Figure 10B); however, no corresponding reduction in H3K18ac could be detected. At 85 h post-transfection the H3K9me3 mark could be observed not just at the NE, but propagated throughout the nucleoplasm (Figure 10C). By contrast to the absence of an effect on acetylation at 21 h, at the later timepoint NET23/STING expressing cells exhibited a strong decrease in H3K18ac staining along with another mark of active chromatin, H3K4 di-methylation [55] (Figure 10C). We also tested for the combination of H3K9me3 and S10 phosphorylation, which is a particularly interesting mark because it is associated with polycomb repressed genes and adds to them a higher level of repression [56]. This mark was reduced in the NET23/STING expressing cells indicating a specific loss of repression at polycomb marked genes; so the changes in epigenetic marks include both a general increase in H3K9me3-associated silencing and some genes being loosened from particularly strong repression.

**Figure 11:**
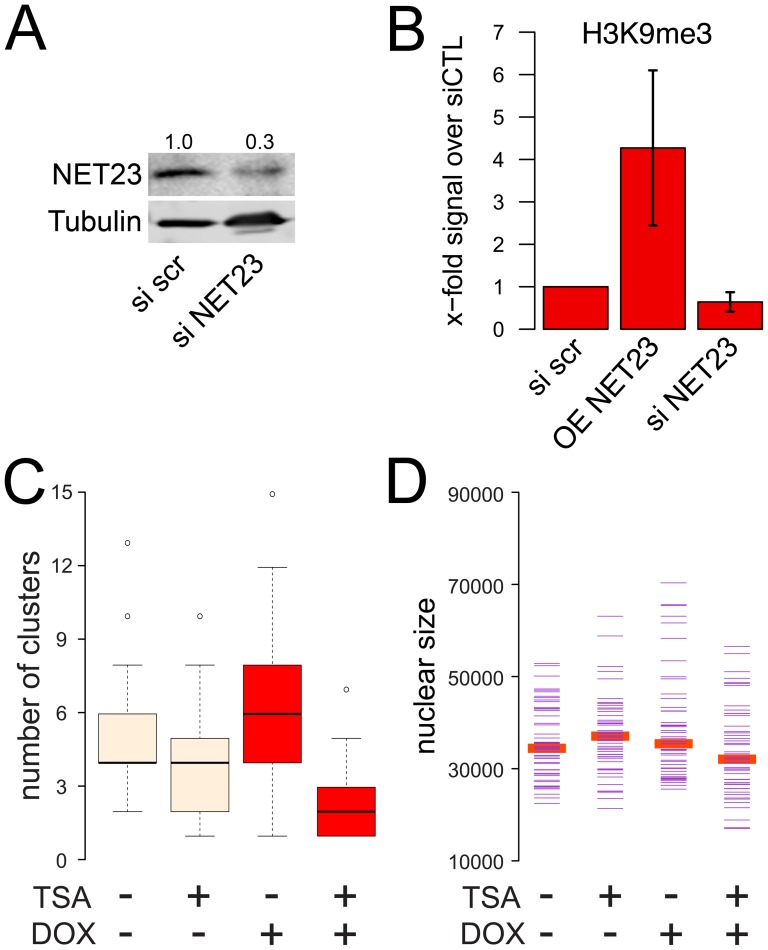
Modulation of NET23/STING levels changes levels of epigenetic marks and the chromatin compaction is reversible by treatment with TSA, a deacetylase inhibitor. (**A**) Knockdown of NET23/STING. HT1080 cells were treated with either siRNA oligos for NET23/STING or a scramble control siRNA oligo. With this treatment NET23/STING protein levels could be reduced to 30% of initial levels at 4 d post-transfection. (**B**) Cell lysates were generated from a population of HT1080 cells either treated with the scramble control or NET23/STING siRNAs or a stably-transfected HT1080 line induced to express NET23/STING with doxycycline. Staining for the total levels of the H3K9me3 mark in these populations revealed that overall levels of H3K9 methylation were increased roughly 4-fold by exogenous expression of NET23/STING while overall levels appeared to be slightly reduced in the NET23/STING knockdown cells. The average from 3 experiments is shown with standard deviations. (**C–D**) The stably-transfected inducible NET23/STING cell line was either not treated or treated with 1 µg/ml of the histone deacetylase inhibitor TSA with or without induction of exogenous NET23/STING by doxycycline (DOX). (**C**) The number of high-intensity pixel clusters measured with the unbiased chromatin compaction algorithm is shown. NET23/STING induction increases the number of clusters while TSA completely reverses this effect. (**D**) Nuclear size was also quantified, revealing that neither doxycycline nor TSA treatment yielded any noticeable effect on nuclear size.

The immunofluorescence staining was performed on transiently transfected cells to avoid contributory effects from cell selection; however, in the intact cells it is possible that epitope accessibility within the compacted chromatin might influence the results. Moreover, transient transfection of plasmid DNA could induce innate immune responses to foreign DNA and so could complicate distinguishing NET23/STING direct effects from downstream effects of innate immune response signaling. Therefore, a stable doxycycline-inducible cell line expressing NET23/STING was generated. Lysates were prepared from this line after 72 h of induction and also from the parent line with a control siRNA or a NET23/STING siRNA knockdown after 72 h (siRNA treatment has been found to not induce innate immune responses). Overall levels of H3K9me3 were increased roughly 4-fold on average by NET23/STING overexpression and reduced slightly by its knockdown as measured with Western blot quantification using fluorescent antibodies (Figure 11A and B).

Treatment with the histone deacetylase (HDAC) inhibitor trichostatin A (TSA) results in genome-wide hyperacetylation with concomitant reversible chromatin decondensation [57]. To test if the compaction induced by NET23/STING is reversible, the stable inducible line was treated with TSA with or without induction of the NET23/STING with doxycycline. Thus stably transfected cells carrying the inducible NET23/STING-GFP fusion were induced with doxycycline for 48 h to promote chromatin compaction and then treated for 6 h with TSA. In uninduced control cells expressing endogenous levels of NET23/STING the TSA noticeably reduced the amount of chromatin compaction measured with the cluster algorithm, though p-values just missed the threshold of significance at p = 0.0578 (Figure 11C). Induction of the exogenous NET23/STING-GFP fusion with doxycycline as before caused a notable increase in high intensity pixel clusters, though as the stable inducible system yielded weaker expression than transient transfections the p-value was only p = 0.0069. Treatment of the doxycycline-induced cells with TSA fully reversed the increased chromatin compaction achieved in the NET23/STING induced cells, fully reducing it to even slightly lower levels than those achieved by the TSA in the uninduced cells with p<2.0E-16 (Figure 11C). This effect was independent of changes in nuclear size (Figure 11D). Thus all chromatin compaction achieved by NET23/STING expression is reversible with TSA.

**Figure 12:**
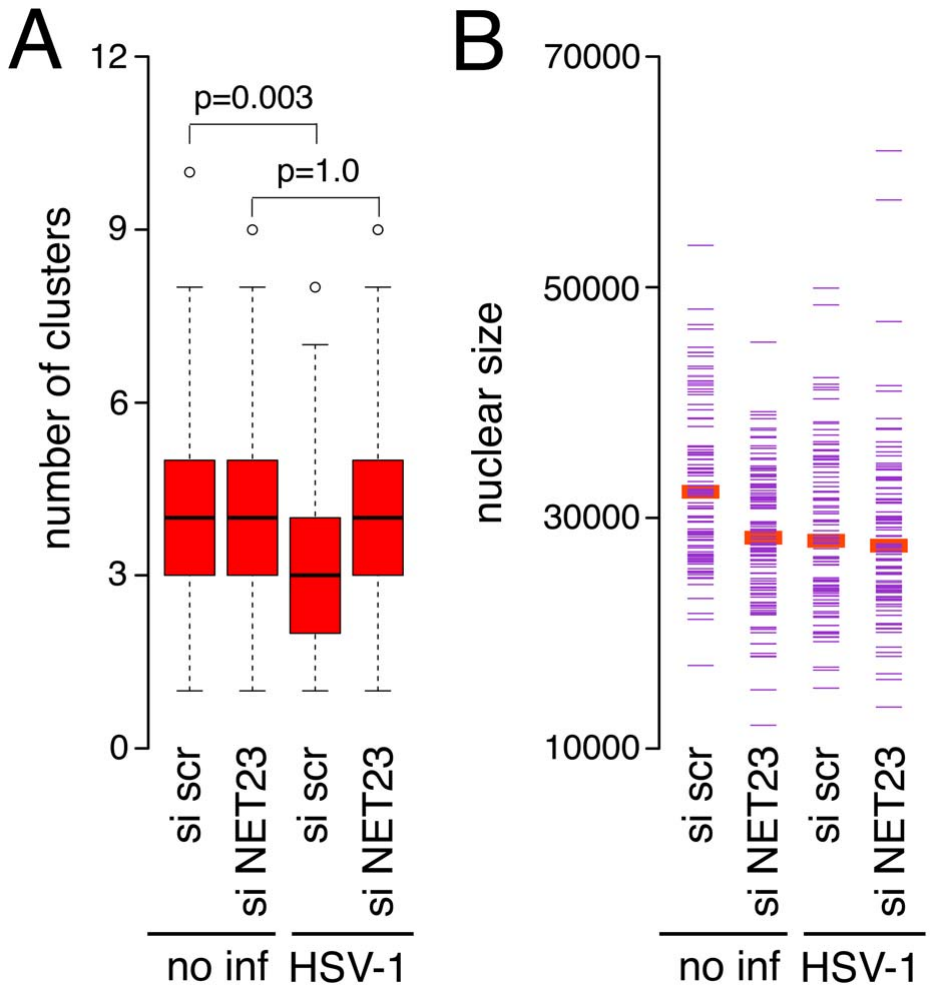
Effect of NET23/STING knockdown on chromatin changes in HSV-1 infected cells. (**A**) Three days after control siRNA or NET23/STING siRNA treatment to deplete NET23/STING protein levels as in Figure 11, cells were infected with HSV-1 for 2 h at MOI 5 to induce innate immune responses. The cells were fixed, stained with DAPI and analyzed with the cluster algorithm. P-values using KS tests to compare the HSV-1 infected cells between the NET23/STING conditions are given. The p value for comparing the two HSV-1 infected populations is p<0.001. (**B**) Analysis of nuclear size in the same populations indicated some differences in nuclear size in this experiment. More than 100 cells were analyzed for each condition for all parameters.

As a role for NET23/STING has been clearly indicated in innate immune response signaling, we wondered if stimulation of innate immune responses with herpes simplex virus-1 (HSV-1) infection would affect the compacted state of chromatin as measured by the cluster algorithm and if any such effects could be mediated by NET23/STING. Thus HT1080 cells were either treated with the control scramble siRNA or NET23/STING siRNA for three days to knock down NET23/STING prior to infection with HSV-1 for 2 h to induce the innate immune response. As the effect of NET23/STING overexpression was much greater than the effect of the knockdown on H3K9me3 staining (Figure 11) and HT1080 cells have little compacted chromatin to begin with, it is not surprising that no discernible effect for NET23/STING knockdown was observed with the cluster algorithm. However, NET23/STING knockdown prevented changes to chromatin compaction that normally occur early upon HSV-1 infection. Interestingly, the HSV-1 infection in the scramble siRNA control HT1080 cells resulted in a reduction rather than an increase in cluster number (Figure 12A, p = 0.003). This reduction was not observed in the cells knocked down for NET23/STING with over 100 cells counted for each condition (Figure 12A, p>0.9) such that the difference between the control siRNA and NET23/STING siRNA knockdown conditions for the HSV-1 infected cells had a p value <0.001. However, nuclear size was also affected slightly in this experiment (Figure 12B) so that it is not possible to unequivocally determine that the chromatin compaction alone accounts for the effects NET23/STING knockdown had on the changes induced by the HSV-1 infection.

## Discussion

NET23/STING was previously linked to functions in innate immune signaling and apoptosis [42], [43], [46], though these two roles were not evidently linked. The role we have shown here for NET23/STING in promoting epigenetic changes and an intermediate chromatin condensation state that is frequently associated with subsequent apoptosis provides a possible means to link these disparate functions.

The goal of a cell in response to pathogen infection is to prevent the propagation of the pathogen within the host organism. A core mechanism to do this is to induce apoptosis; however, many pathogens have mechanisms for interfering with signaling of apoptosis. A way for the cell to get around this and still promote its death along with the pathogen is to activate innate immune signaling for the release of interferons to target the immune system to the infected cell. In this latter process several changes to the epigenetic signature of affected cells have been observed. For example, H3K9 di-methylation at interferon genes themselves correlates inversely with the level of interferon expression and its addition is thought to reflect a host cell mechanism to temper responses in order to avoid inflammatory disease [58]. Although it may at first seem counter-intuitive as the cell needs to robustly express interferons to target immune cells to the infected host cells, silencing epigenetic marks are very important for the global innate immune response as treatment of cells with histone deacetylase inhibitors had overall inhibitory effects on innate immune responses [59]. This is consistent with our finding that NET23/STING in addition to its upstream pathogen sensing activities [60], [61] also contributes to a large-scale change in the overall pattern of epigenetic marks. It remains unclear why the effect of HSV-1 infection in HT1080 cells was to reduce instead of increase cluster number (used as a measure of chromatin compaction), though the nuclear size changes in this experiment could affect the output of the algorithm. Nonetheless, the effect of the virus was mitigated by NET23/STING knockdown.

The pathogens themselves can also target immune response epigenetics in that histone deacetylase expression is found to increase upon infection of plants with pathogens and transgenic plants overexpressing the deacetylase are more susceptible to infection [62]. Thus, there is likely a “tug-of-war” effect going on in the infected cell between the pathogen efforts to block host cell responses and the host cell to find the right balance in its response. Intriguingly, NET23/STING can also go too far when unregulated and its exogenous overexpression can also cause experimental autoimmune encephalitis [63]. The ability of the nuclear membrane pool of NET23/STING to promote chromatin modifications is not only a new function for this extremely multi-functional protein, but also may reflect a creative mechanism for the host cell to get around the efforts of the pathogen to block apoptotic and innate immune responses. Many pathogens target central channel transport through the nuclear pore complex — for example herpesvirus ICP27 protein targets the central channel nucleoporin Nup62 [64] — but NETs can travel through the peripheral channels of the nuclear pore complex [65]–[68]. Thus, even when central channel transport is blocked, NET23/STING should in theory be able to target to the inner nuclear membrane where it could engage its nuclear functions.

Although the finding of epigenetic transmission in response to signaling through the peripheral channels is relatively novel, a NET function in promoting epigenetic changes has been shown for other NETs that promote certain epigenetic marks by binding to silencing factors and recruiting them to the NE. For example the NET LBR can bind DNA methylating enzyme MeCP2 and the NETs LAP2β and emerin can bind the histone deacetylase HDAC3 [33], [36], [69]. The significant increase in H3K9me3 by Western blot and its first appearance at the nuclear periphery by immunofluorescence microscopy in the cells exogenously expressing NET23/STING suggests that this protein may directly recruit histone modifying enzymes. However, future work will be needed to test this.

How NET23/STING can pivot the cell for this chromatin condensation and innate immune response or apoptosis choice will also require many future studies, but this is the first study to our knowledge that demonstrates a nuclear function for this important multi-functional protein. One direction that will be interesting to pursue is what drives the condensation to the point of inducing apoptosis. What can be inferred from this study is that this can occur by both p53 dependent and p53 independent pathways. This is because fusions at either end of NET23/STING induced apoptosis with similar kinetics in the wild-type cells, but the construct with GFP fused at the N-terminus of NET23/STING exhibited a delayed response in the p53^−/−^ cells with more annexin V-positive and fewer PI-positive cells. While this may reflect in part some validity to an earlier report that tags, particularly N-terminal tags, affect its function [42], the fact that this lagging effect was only observed in the p53^−/−^ cells suggests that NET23/STING can act by both p53-dependent and p53-independent pathways and this distinction only becomes apparent when the tag weakens the activity of NET23/STING.

The original motivation for this chromatin compaction screen was to identify proteins that may be involved in mediating the aberrant chromatin distribution pathologies observed with several lamin and NET-linked diseases [3]–[7], [10], [11]. The role we have indicated for NET23/STING in endogenous chromatin compaction suggests that in addition to its functions in innate immunity it also contributes to mediating chromatin distribution patterns in disease. This is further supported by observations that both the NET23/STING compaction phenotype and the observed electron microscopy changes in chromatin in some NE diseases have been linked to epigenetic changes. For example, in fibroblasts from Hutchison-Gilford Progeria syndrome caused by mutation of the NE lamin A protein [70], [71], H3K9me3 and H3K27me3 that are associated with silenced chromatin were reduced while H4K20me that is associated with active chromatin was increased [14]. A potential link between NET23/STING and lamin A, causative of many of these NE-linked diseases, is further suggested by our previous observation that distribution of NET23/STING at the NE was lost in lamin A knockout mouse embryonic fibroblasts [44]. Thus we postulate that in addition to its effects in innate immune signaling NET23/STING may also be involved in some of the chromatin changes that occur in NE diseases.

## Methods

### Plasmid construction

NET expression plasmids used in the screen were cloned from IMAGE collection cDNAs as previously described [41], [44]. Most NETs were fused to monomeric red fluorescent protein (mRFP) at their carboxyl-terminus while a few were fused to an HA epitope tag at their amino-terminus. All those used in the screen were under regulation of the CMV promoter. After its identification in the screen, NET23/STING was additionally cloned into both the pEGFP-N2 and pEGFP-C2 vectors for C- and N-terminal GFP fusions. The pEGFP-N2 fusion was further subcloned into pLVX-TRE3G using NheI and NotI as restriction sites for subsequent generation of lentiviruses for transduction to make stable inducible cell lines.

### Cell culture, generation of stable lines, transfection and HSV-1 virus infection

HeLa cells stably transfected with H2B-GFP ([72]), HT1080 human fibrosarcoma cells (ATCC, CCL-121), MRC5 normal human fetal lung fibroblasts (ATCC, CCL-171), 216−/− lamin A knockout mouse embryonic fibroblasts [73], U2OS human bone osteosarcoma cells (ATCC, HTB-96), HepG2 human liver carcinoma cells (ATCC, HB-8065), HEK/293T human embryonic kidney cells (ATCC, CRL-11268), NIH3T3 mouse fibroblasts (ATCC, CRL-165), BJ foreskin fibroblasts (ATCC, CRL-2522), AG short for AG-08470 dermal skin fibroblasts from a 10 year old normal female (Coriell Institute for Medical Research, AG-08470), HCT116 human colon carcinoma cells and their p53^−/−^ knockout variant 379.2 (kind gift of B. Vogelstein, Johns Hopkins University; [74]) were maintained in high glucose DMEM (Lonza) supplemented with 10% fetal bovine serum (FBS), 100 µg/µl penicillin and 100 µg/µl streptomycin sulfate. To maintain the stable transfection of H2B-GFP these cells were treated with 100 µg/ml geneticin every other passage. Jurkat and EL4 cells, human lymphocyte cell lines, were cultured in RPMI with 10% FBS and antibiotics.

To generate a stable inducible NET23/STING expressing cell line, lentiviruses encoding a doxycycline inducible NET23/STING fused to GFP at the C-terminus were prepared by standard procedures and transduced onto HT1080 cells. Transduced cells were selected with geneticin at 500 µg/ml.

Cells were plated on coverslips at ∼10% confluency to prevent their reaching confluency before fixation at 40 h post-transfection. DNA was transfected 12 h after plating using Fugene 6 (Roche) according to the manufacturer's instructions. Inducible stable lines were induced with 1–2 µg/ml doxycycline and for reversal of chromatin condensation cells were treated with 1 µg/ml TSA.

For HSV-1 virus infection HT1080 cells were plated onto a 6 well plate (∼200.000 cells/well). Cells were infected with a multiplicity of infection (MOI) of 5 using HSV-1 wild type virus. The virus was added to cells in 0.5 ml culture medium. The cells were incubated for 1 h at 37°C and 5% CO2 to facilitate the adsorption of the virus. Subsequently 1.5 ml medium were added and the cells were incubated for additional 2 h before cell fixation and analysis.

### Screen for NETs that alter chromatin organization

NETs fused at their C-termini to mRFP or at their N-termini to the HA epitope tag were transfected into H2B-GFP cells (kind gift of G. Wahl, Salk Institute; [49]) and fixed between 48 and 60 h post-transfection. Coverslips were directly mounted onto slides in Fluoromount G (EM Sciences) and imaged.

### Antibodies

To stain for NET23/STING, Tmem173 polyclonal antibody (ProteinTech, 19851-1-AP, concentration 59 µg/150 µl) was used at a 1/400 dilution. To stain for epigenetic marks rabbit antibodies recognizing H3K18Ac (Ab1191, Abcam 1∶500), H3K4Me2 (Ab7766 Abcam 1∶500), H3K9me3 (07-523, Upstate 1∶200), and the combination of H3K9me3 and H3S10 phosphorylation (Ab5819, Abcam 1∶500). Lamin antibodies were previously described in [75]. All fluorophore-conjugated secondary antibodies used for immunofluorescence were minimally cross-reactive from donkey (Jackson ImmunoResearch) or goat (Molecular Probes). For Western blotting IR800 conjugated goat anti-rabbit antibodies (LI-COR Biosciences) were used.

### Live cell imaging

Live cell imaging was performed on a Leica SP5 laser scanning confocal (Leica Microsystems). H2B-GFP was visualized with the 488 nm line of an Argon laser with excitation filter settings of 495–550 nm. NET23-RFP was visualized using a 561 nm diode laser with excitation filter settings of 570–630 nm. For all imaging the lasers were set as low as possible to prevent photo-toxicity and bleaching. The cells were cultured on #1.5 25 mm round coverslips (Warner Instruments) and transferred to the Attofluor Chamber (Invitrogen), which was mounted on the confocal. Cells were then maintained at 37°C and 5% CO_2_ for the duration of the experiment.

### Immunofluorescence microscopy

Cells were fixed for 7 min in 3.7% formaldehyde, permeabilized for 6 min in 0.1% Triton X-100, blocked with 4% BSA in PBS, and reacted for 40 min at RT with primary antibodies for histone modifications. After washing, appropriate fluorophore-conjugated secondary antibodies were added for 30 min at RT and washed. Cells were also stained with DAPI (4,6-diamidino-2 phenylindole, dihydrochloride) to visualize nuclei and mounted in fluoromount G (EM Sciences).

Most images were obtained using a Nikon TE-2000 microscope equipped with a 1.45 NA 100× objective, Sedat quad filter set, PIFOC Z-axis focus drive (Physik Instruments), and CoolSnapHQ High Speed Monochrome CCD camera (Photometrics) run by IPLab image acquisition software. Image stacks (0.2 µm steps) were deconvolved using AutoquantX. High-resolution images were taken using a Deltavision (Applied Precision) microscope system with 100×1.4 NA objective and 0.2 µm stacks were deconvolved using DeconQ and images were processed using SoftWorks. Micrographs were saved from source programs as.tif files and prepared for figures using Photoshop 8.0.

### Image quantification of chromatin compaction

All images were captured using Metamorph acquisition software with identical settings after identical staining and fixing conditions. At least 50 nuclei were analyzed per condition. To distinguish individual nuclei in a field, nuclei were either thresholded or manually identified and segmented with individual masks. Pixel intensities were extracted from raw 16-bit images in TIFF format as a numerical matrix in Image J 1.33, and subsequent analysis performed in R (http://www.R-project.org). Raw pixel intensities were normalized to the sum of total number of pixel intensities in each nucleus, to account for possible differences in overall intensity between nuclei, and localized peaks of higher signal, corresponding to denser chromatin, were identified by taking the 15^th^ percentile of the normalized pixel signals as a lower threshold. The resulting signal peaks were then filtered so that peaks smaller than 20 pixels were discarded, and peaks closer than 3 pixels were joined together. Images taken with the microscope configuration described in the *Immunofluorescence microscopy* section above correspond to 1 pixel equaling 0.0645 µm. The distribution of numbers and areas of each individual peaks were then calculated and compared between samples using Kolmogorov-Smirnov tests for statistical significance.

### Quantification of apoptosis and chromatin state by flow cytometry

Plasmids encoding NET23/STING fused to GFP at the N- or C-terminus (GFP-NET23 and NET23-GFP respectively), NET23-mRFP or the fluorescent proteins alone were transfected into HT1080 cells or HCT116 cells and the p53 knock out variant 379.2 (p53^−\−^) of HCT116 cells using Fugene HD transfection reagent (Roche). In experiments where apoptosis was blocked, immediately after transfection cells were treated with 20 µM Z-VAD, a pan-caspase inhibitor, from a 10 mM stock in 30% DMSO. At 48 h post-transfection and Z-VAD treatment, the DNA stain Hoechst 33342 (Molecular Probes) was added to the cells at a final concentration of 5 µg/ml and left to incubate at 37°C for a period of 30 min to 60 min. Cells were harvested by trypsinization followed by its inactivation with serum, cell pellets were collected by centrifugation at 250×g for 5 min at RT, washed once in PBS and re-suspended in 1 ml of PBS. For apoptosis analysis the cell pellet was re-suspended in 1 ml of Annexin binding buffer (BD biosciences), treated with RNase A, and 2.5 µg/ml of Annexin V-Alexa flour 647 was added. Cells were incubated in the dark at room temperature for 5 min. Cells were kept on ice and Propidium Iodide (50 µg/ml) was added together with RNase (100 µg/ml). Cells were immediately analyzed on an LSR II flow cytometer (BD Bioscience, UK) equipped with 488 nm and 350 nm lasers and appropriate filters. Cellular debris and cell aggregates were excluded from analysis by application of electronic gates. Cell cycle analysis was carried out on the live singlets gate using *FlowJo* software (TreeStar, Inc). At least 10,000 cells were scored for the total live singlets and 1,000 cells for the GFP or mRFP positive live singlets. Each experiment was repeated at least 2 times. Data are displayed in the form of histogram overlays using %Max option, which scales each population curve to mode = 100%.

### siRNA knockdown of *NET23/STING*

NET23/STING siRNA oligos “GCACCUGUGUCCUGGAGUAUU” and “GCAUCAAGGAUCGGGUUUAUU” were combined with 0.72 µg each or a control scrambled sequence were transfected into cells using JetPrime transfection reagent. To confirm knockdowns, cell lysates were prepared and levels tested by Western blot with NET23/STING antibodies.

### Western blotting

To increase lamina solubility, Jurkat cells were incubated on ice in 50 mM Tris-HCl pH 7.4, 150 mM NaCl, 2 mM MgCl_2_, 0.2% NP-40 in the presence of protease inhibitor cocktail (Roche 11 873 580 001) 15 min, then sonicated in a 4°C sonibath, followed by adding sample buffer (100 mM Tris pH 6.8, 4 M Urea, 2% SDS, 50 mM DTT and 15% sucrose), heating at 65°C and then sonicating again in a 4°C sonibath. Equal numbers of cells were loaded between the wild-type cells and the NET23/STING knockdown cells. Equal protein loading was confirmed by coomassie blue staining of polyacrylamide gels. Proteins were resolved by SDS-PAGE and transferred to Nitrocellulose membrane (LI-COR Biosciences). Membranes were blocked in PBS, 6% milk, 0.2% tween. For blots probed for H3K9me3 to ensure that histones were not lost Immobilon 0.2 µm pore size membranes (ISEQ10100) were using only a 45 min transfer time. Primary antibodies were diluted in the PBS-milk-tween buffer and allowed to incubate overnight at 4°C. Secondary antibodies IR800 conjugated goat anti-rabbit (LI-COR Biosciences) were added at concentration 1/5000 at RT for 2 h. Visualization and quantification were performed using a LI-COR Odyssey and software (Odyssey 3.0.16) using median background subtraction. Three independent blots were run for each experiment and averages from all three are presented.
